# Supplementary material for: Are carbon emissions trading and green financial instruments synergistic? -Comprehensive quantitative research based on content analysis
Source: PLoS One. 2024 Mar 7;19(3):e0298601. doi: 10.1371/journal.pone.0298601 (PMC10919601; doi:10.1371/journal.pone.0298601)
Supplement: S1 Data — (ZIP) [file pone.0298601.s001.zip › Data/地级市Z_F工作报告.zip!/╜¡╬≈/╙Ñ╠╢╩╨/└┤╫╘╣1⁄2╓┌║┼í╛┬φ┐╦╩2╛▌═°í┐_2016.docx]

各位代表：

现在，我代表市人民政府，向大会作政府工作报告，请予审议，并请各位市政协委员和列席会议的同志提出意见。

一、2015年及“十二五”工作回顾

2015年是完成“十二五”规划的收官之年，是我们适应、把握、引领新常态的重要一年。在省委、省政府和市委的正确领导下，全市上下认真学习贯彻习近平总书记系列重要讲话特别是“一个希望、三个着力”指示精神，紧紧围绕省委十六字方针，按照“主攻项目、决战‘三区’，凸现特色、实现跨越”总体要求，坚持把稳增长、促改革、调结构、惠民生作为首要任务，全力落实稳增长促发展40条和创新创业36条具体举措，大力推进“8+1”重点工作，经济运行总体平稳、稳中有进。预计(下同)全年完成生产总值640亿元、增长9%，财政总收入112.4亿元、增长10.6%，一般公共预算收入83亿元、增长 13.1%，规模以上工业增加值350亿元、增长9%，固定资产投资532亿元、增长14.5%，社会消费品零售总额168亿元、增长10.2%，城镇居民人均可支配收入26930元、增长9.5%，农村居民人均可支配收入12485元、增长10%。

(一)产业升级提质增效。沿320国道产业集聚升级带建设加快推进。电镀集控区、小微企业孵化基地一期、总部经济大楼等项目陆续建成，建成工业重点项目22个，新增产值150亿元，鹰潭高新区、贵溪经开区跻身全省工业园区十强。铜产业转型升级步伐加快。众鑫成年产2万吨铜基复合线材等7个项目开工建设，红旗铜业12万吨高性能铜材等8个项目建成投产，铜精深加工占比提升8个百分点。成功承办中国(鹰潭)“互联网+铜产业”发展暨2015年铜交易大会，内陆首家铜期货交割仓库和中江国际商品交易中心开库运营，铜现货贸易量达40万吨。地方特色产业加速集聚。养元“六个核桃”等14个非铜产业项目竣工投产，水工、节能照明主营业务收入分别增长10%、12%，新能源光伏发电项目装机容量达100兆瓦，非铜产业增加值占规上工业比重提升2.8个百分点。旅游产业不断壮大。全省旅游发展大会筹备工作进展顺利，圣井山综合开发等21个重点旅游项目有序实施，寻梦龙虎山、地质花卉公园市场反响热烈，全市接待国内游客增长38%，旅游总收入增长45%，我市列入首批国家级旅游业改革创新先行区，龙虎山景区获批国家可持续发展实验区。商贸物流业快速发展。十大专业市场开业运营5家、进驻商户510余家，实现营业额5.8亿元。货运业实现销售收入60.49亿元、上缴增值税4.97亿元，现代物流园被评为全国优秀物流园区。电子商务产业实现交易额270亿元、增长123%，高新区获批全省首家跨境电商产业示范基地。现代农业稳步发展。粮食总产达70万吨。白鹤湖、雷溪、杨溪现代农业示范园区进驻龙头企业29家，全市新增农民专业合作社516家、家庭农场112家，179家市级以上农业龙头企业实现销售收入82亿元、增长11%。

(二)新型城镇化加速推进。国家新型城镇化综合试点和省级“多规合一”试点建设稳步推进，市城乡总体规划暨“多规合一”(2014—2030年)和新型城镇化规划(2014—2020年)编制基本完成。320和206国道拓宽改造、余信贵大桥、余信贵大道等城际交通项目稳步推进，新二中、恒大学校、人民医院信江新院、信江菜市场、信江污水处理厂等新区配套项目进展顺利，城南污水处理厂、生活垃圾焚烧发电厂、排水排污管网等老城改造项目有序实施。盐业公司、磷肥厂等棚户区和城中村改造有序推进，全市棚改项目开工率100%，完成棚改60万平方米。采取降低住房公积金贷款门槛、“以购代建”、商品房抵扣等政策稳定住房消费，房地产市场逐步回暖。深化户籍制度改革，健全城镇基本公共服务体系，新增城镇常住人口2.45万人。十强中心镇标准化建设和扩权强镇试点稳步推进，187个秀美乡村建设点全面完成。深入开展城乡环境专项整治，全面推进大气、水、土壤污染防治三大战役，实施PM2.5监测，农业面源污染、水库水质治理成效明显，11个中小河流治理项目基本完工，我市列入国家城乡生活垃圾委托第三方治理试点城市，贵溪、余江获省级森林城市称号。

(三)重点改革取得突破。加快政府职能转变。“三单一网”建设顺利推进，政府部门权责清单公布实施，全面清理非行政许可审批事项，145家中介服务机构入驻“中介超市”，全市各类公共资源实现网上交易。政府购买公益性岗位试点顺利推进。“12345”政务服务热线试运行。市县两级不动产统一登记中心挂牌成立。公务用车改革扎实推进。深化财税金融改革。组建四大市级融资平台，改革重组市投资集团。市工业控股有限公司和中小企业信用担保有限公司开业运营，设立企业应急转贷基金，累计为企业解决融资15亿元。“财园信贷通”“财政惠农信贷通”发放贷款9.5亿元。江西银行、九江银行在鹰设立分支机构，全省首个小贷资产证券化项目在上海证交所挂牌，3家企业在“新三板”、5家企业在上海股交中心挂牌。盘活财政存量资金近20亿元，争取地方政府债券额度34.8 亿元，其中置换存量债务27.1亿元。推进农村综合改革。余江国家农村宅基地制度改革试点工作稳步推进，圆满承办全国宅改集中调研交流会。农村土地承包经营权确权登记颁证工作基本完成，农村土地流转率38.6%。完成国有林场改革。

(四)发展后劲明显增强。项目建设加快推进。实施大中型建设项目345项，完成投资292亿元。向社会发布总投资430亿元的59个PPP试点项目。争取国家专项建设基金项目41个、资金16.35亿元。高新区9亿元企业债券成功发行。贵溪、余江列入全国深化县城基础设施投融资体制改革试点，鹰潭机场、鹰潭港、信江三级航道等项目纳入省“十三五”规划。全方位开放力度加大。全力对接“民企入赣”，市直八大领域引进项目122个，鹰潭高新区总投资40 亿元的十大项目集中开工，全市引进省外5000万元以上项目实际进资278亿元、增长13.6%，实际利用外资2.4亿美元、增长11.3%。关检合作 “三个一”模式顺利实施，开通宁波至鹰潭转检通道，实现外贸出口9.3亿美元、增长4.8%。创新创业步伐加快。铜检中心通过国家“三合一”验收现场评审，铜产业工程技术研究中心取得省级资质，高新区获批国家知名品牌创建示范区，全年新增高新技术企业21家。深化商事制度改革，实行“三证合一、一照一码”，新登记企业3154家、增长27.9%。

(五)民生事业持续改善。一般公共预算支出中民生支出占70%，十大惠民工程75件实事基本完成。新增城镇就业2.48万人，城镇就业率97.3%，新增转移农村劳动力1.77万人。连续11年调整企业退休人员养老金，月人均增资199元，为5.8万企业离退休人员发放养老金12.1亿元。全市3万余名被征地农民纳入城镇职工养老保险。“多险合一”实现各项社保信息“同人同城同库”管理，城乡居民养老保险参保率95%以上。启动实施机关事业单位养老保险制度改革。新农合参合率99.1%。城乡低保标准分别提高到每人每月450元、240元。新建1200套公租房全部开工。大力实施精准扶贫“六大工程”，全年减贫7365人。江西师范高等专科学校和鹰潭卫生学校获批，中等职业教育“三校合一”整合工作加快推进。国家城市公立医院综合改革试点全面启动，村级卫生计生服务室标准化建设实现市域全覆盖。成功举办对台千人专场授箓活动和第八届海峡两岸道教文化论坛。启动实施第六届优秀政府文艺作品奖评选活动。大上清宫遗址考古发掘获国家文物局批准。全民健身活动精彩纷呈，承办了全国乒超联赛八一女队主场比赛和环鄱阳湖国际自行车赛。

余江县荣获全省征兵工作模范县称号，“双拥”工作取得丰硕成果。食品药品安全、安全生产和治安形势持续平稳，社会保持和谐稳定。统计、档案方志、民族宗教、外事侨务、气象、防灾减灾、人防民防、妇女儿童、老龄、残疾人等工作取得了新的成绩。

(六)政府自身建设不断加强。扎实开展“三严三实”专题教育，严格落实“一准则两条例”、党风廉政建设“两个责任”和中央八项规定，开展违规插手工程建设项目和教育、医疗、殡葬、税收四大领域“红包问题”等专项治理，加大审计监察和查处腐败案件力度。自觉接受市人大法律监督和市政协民主监督，办复人大代表建议69件、政协提案115件，代表、委员满意率100%。实施法治政府建设考核评价，市行政复议委员会成立并运行，市县政府和城区40个社区全部建立法律顾问制度。强化政府部门绩效考核和行政问责，电子政务综合办公平台正式运行，政务环境进一步优化。

需要向代表报告的是，随着我国经济发展进入新常态，增速由高速向中高速转变，“十二五”后期增速普遍放缓，我市经济面临前所未有的下行压力。2015 年主要指标中，工业增加值、固定资产投资、社会消费品零售总额实际增速比年初预期目标分别低2、1.5、2.8个百分点左右。对照“十二五”规划目标，部分指标略低于预期目标，其中生产总值、实际利用外资分别低1.3、0.2个百分点左右。总体看，我市“十二五”规划目标任务基本完成，经济社会发展取得了新的成就。

——这五年是我市经济总量稳步扩大、综合实力显著增强的五年。全市生产总值是“十一五”末的1.7倍，人均生产总值达8740美元，较“十一五”末增长62.2%。财政总收入突破100亿元大关，较“十一五”末实现翻番，一般公共预算收入是“十一五”末的2.8倍。固定资产投资五年累计达1987亿元，是“十一五”期间的2.9倍。社会消费品零售总额、出口总额分别比“十一五”末增长94%、156%。

——这五年是我市产业结构不断优化、发展动能加快转换的五年。三次产业结构由9.5:62.8:27.7调整为7.6：60:32.4。规模以上工业增加值是“十一五”末的1.8倍，市属工业增加值占全市规上工业比重突破60%。“七位一体”铜产业体系基本形成，铜产业循环经济基地获批国家“城市矿产”示范基地，铜产业主营业务收入较“十一五”末实现翻番。水工、节能照明、大健康、眼镜等特色产业产值实现四年翻番。鹰潭高新区升格为国家高新技术产业开发区。龙虎山跻身国家5A级旅游景区，新增4个国家3A级旅游景区，成功承办第三届国际道教论坛。现代物流园、国际商贸园初具规模，集聚发展态势日益显现。农业综合生产能力显著增强，成功创建贵溪国家级现代农业示范区。

——这五年是我市城乡面貌深刻变化、鹰潭魅力明显提升的五年。全市建成区面积由58.9平方公里拓展到71.2平方公里，城镇化率达55.53%，较 “十一五”末提高8.13个百分点。信江新区30平方公里内的主干路网全面贯通，建成区面积达8平方公里，“一江两岸”城市格局已经形成。完成棚户区和城中村改造1.41万户，完成城市30余条主次干道和老城区85%的小街小巷提升改造。建成信江两岸十里绿色景观长廊，城区绿化面积138万平方米，成功创建国家园林城市。镇村联动建设扎实推进，塘湾、锦江、罗河、上清列为国家重点镇，完成“秀美乡村”建设点1151个。荣获全国国土资源节约集约模范市称号。

——这五年是我市改革开放深入推进、后劲活力持续增强的五年。余江农村宅基地制度、城市公立医院、行政综合执法体制、智慧城市、信息惠民等列入国家改革试点，一系列关键领域的改革取得突破。开放型经济成果丰硕，累计引进省外5000万元以上项目实际进资1047.2亿元、年均增长19%，累计利用外资 9.59亿美元、年均增长14.8%，连续五年被评为全国外贸百强城市。新增省级以上工程技术研究中心9家、企业技术中心7家，国家、省级科技立项253 项，科技进步对经济增长的贡献率提高5个百分点。

——这五年是我市社会事业全面进步、人民群众得到更多实惠的五年。城乡居民人均可支配收入分别比“十一五”末增长72.4%和99.8%。五年新增城镇就业13.7万人，转移农村劳动力29.8万人。累计建成保障性住房6.2万套，中心城区保障性住房覆盖率达25%，居全省前列。解决农村饮水不安全人口28.7万人。社会养老保险和“三张网”医疗保险体系均实现城乡居民全覆盖。生态环境进一步改善，森林覆盖率达58.2%，环境空气质量优良率保持在 80%以上，主要河流断面水质、城市集中饮用水水源地水质达标率均为100%，“十二五”减排任务全面完成。平安鹰潭、法治鹰潭建设扎实推进，“六五”普法规划全面完成。

各位代表!五年的不懈拼搏，为鹰潭与全国全省同步全面建成小康社会奠定了坚实基础。展望未来，我们已经站到了新的发展起点上，这必将进一步激励我们信心百倍、豪情满怀地开创鹰潭更加美好的明天!

过去五年的成绩来之不易，是省委、省政府和市委正确领导的结果，是市人大法律监督、市政协民主监督的结果，是全市上下团结一心、奋力拼搏的结果。在此，我代表市人民政府，向全市广大干部群众，向各位人大代表、政协委员、各民主党派、工商联、无党派和社会各界人士，向驻鹰单位、人民解放军、武警官兵、公安民警和离退休老同志，向所有关心、支持、参与鹰潭经济社会发展的各界人士，表示衷心的感谢并致以崇高的敬意!

当前我市经济社会发展还存在不少矛盾和问题，主要表现在：产业层次整体水平不高，特色产业优势仍未得到充分发挥，产业结构调整任重道远;城乡基础设施还不完善，教育、医疗、养老等公共服务与群众需求还有差距;思想解放的力度、开放包容的环境、服务发展的水平等方面还存在不足，服务型政府建设还需进一步加快。对此，我们一定认真对待，切实加以解决。二、“十三五”发展的指导思想和目标任务

“十三五”时期是全面建成小康社会的决胜阶段，也是必须紧紧抓住并可以大有作为的重要战略机遇期。市政府根据《中共鹰潭市委关于制定国民经济和社会发展第十三个五年规划的建议》，编制了《鹰潭市国民经济和社会发展第十三个五年规划纲要(草案)》(以下简称《纲要(草案)》)，提出了今后五年经济社会发展的指导思想和奋斗目标。

指导思想是：高举中国特色社会主义伟大旗帜，以马克思列宁主义、毛泽东思想、邓小平理论、“三个代表”重要思想、科学发展观为指导，深入贯彻习近平总书记系列重要讲话精神，遵循“四个全面”的战略布局，坚持发展第一要务和创新、协调、绿色、开放、共享五大发展理念，紧紧围绕提前翻番、同步小康的总目标，以提质、增效、升级为中心，以创新、改革、开放为动力，按照省委十六字方针，全面落实“主攻项目、决战‘三区’，凸现特色、实现跨越”的总体要求，奋力开创鹰潭“世界铜都、中华道都、田园新城”的新局面，为建设富裕秀美宜居和谐鹰潭打下更加坚实的基础。

《纲要(草案)》提出了“十三五”时期鹰潭市经济和社会发展的总体目标：小康社会全面建成，经济总量和城乡居民收入提前翻番，产业转型升级成效明显，生态环境巩固提升。

——经济规模更加壮大。地区生产总值年均增长8.5%左右，到2020年，达到980亿元;固定资产投资年均增长13%左右，达到1000亿元;财政总收入年均增长10%左右，达到180亿元;社会消费品零售总额和外贸出口总额年均增长10%和2%左右，分别达到273亿元和10.3亿美元，实际利用外资五年累计超过15亿美元。

——转型升级更加明显。产业结构不断优化，三次产业结构调整为5:57:38，旅游业综合收入达到350亿元;科技对经济发展的贡献率不断提高，高新技术产业增加值占规模以上工业增加值的比重达到25%，研究与试验发展经费支出占GDP的比重达到4%;亿元生产总值生产安全事故死亡率低于0.07%。

——人民生活更加富裕。城市布局得到优化，设施功能不断完善，新型城镇化加速推进，城乡一体化发展更加均衡，城乡差距明显缩小，到2020年，全市城镇化率达到62%;城镇居民和农村居民人均可支配收入年均增长均达到9%左右，分别达到41430元和19210元，中等收入人口比重上升;现行标准下的贫困人口全部实现脱贫。

——社会发展更加和谐。就业较为充分，到2020年新增城镇就业人数达10万人，收入差距进一步缩小;社会保障机制更加完善，城乡三项医疗保险参保率达到97%以上，每千人拥有卫生技术人员数达到5.9人;义务教育巩固率达到99%以上，基本实现教育现代化;居民基本医疗保险人数、城镇保障性安居工程建设等完成省政府下达指标。

——生态环境更加秀美。全市森林覆盖率稳定在58.2%左右，森林蓄积量达到850万立方米，主要河流监测断面100%稳定达到或优于Ⅲ类水质;单位GDP能耗年均下降2.7%，累计下降13%;单位生产总值二氧化碳排放量、主要污染物排放量控制在省政府下达的指标范围内，力争AQI良以上等级天数比例稳定在85%以上;城镇生活污水集中处理率、城镇生活垃圾无害化处理率分别提高到95%以上和100%;努力争创国家森林城市和省级生态文明先行示范区。

——改革开放更加深入。在政府职能转变、激发市场活力、构建开放型经济、创新发展机制等重要领域和关键环节上取得明显进展，体制活力和发展动力进一步增强，部分领域成为我省深化改革的试验、示范区;对外开放的广度和深度不断拓展，在加快融入“一带一路”战略、加快国际产能合作方面取得新突破，部分行业走在全省前列。

——法治建设更加完善。法治鹰潭建设取得显著成效，人民民主更加健全，人权得到切实保障，产权得到有效保护，司法公信力明显提高，治理体系和治理能力现代化取得重大进展。

为实现上述目标，《纲要(草案)》还提出了产业转型升级、统筹城乡均衡发展、实施创新驱动战略、加强基础设施建设、大力推进绿色发展、全面深化改革开放、大力增进民生福祉、全面推进依法治市等8个方面的任务和建立健全机制这一保障。指导思想、奋斗目标和主要任务是在正确把握经济发展新常态，认真研究国家和省重大决策部署，全面分析我市各种有利条件，深入查找存在问题与差距的基础上提出的。这些目标任务对于加快我市经济社会发展不仅是必须的，而且通过努力也是完全可以实现的。我们坚信，有省委、省政府和市委的正确领导，有全市上下的团结拼搏，一定能够铸就“十三五”发展的新辉煌!鹰潭人民的生活一定会更加美好!三、2016年的工作安排

2016年是“十三五”开局之年，是全面建成小康社会决胜阶段的第一年。总体看，今年经济下行的压力仍将持续，面临的发展形势更为严峻。我们要进一步增强发展信心，积极应对挑战，确保完成本届政府确定的目标任务，实现“十三五”良好开局。

今年全市经济社会发展主要预期目标是：生产总值增长9%左右，财政总收入增长9%，一般公共预算收入增长9%，规模以上工业增加值增长9%，固定资产投资增长15%左右，社会消费品零售总额增长11%，城镇居民人均可支配收入增长9%，农村居民人均可支配收入增长10%，居民消费价格总水平涨幅控制在 3%左右，完成省下达的节能减排计划任务。

实现上述目标，必须在新常态下进一步解放思想，创新举措，既要坚持稳增长、调结构、惠民生、防风险，更要积极寻找新的发展动力源，加快培育新的经济增长点。主要是把握“五个更加注重”：一是更加注重供需结合，增强发展动能。坚持从供给侧和需求侧两端发力，通过在基础设施、棚户区改造、公共配套等领域充分挖掘内需潜力，保持有效投资规模，不断优化需求结构;大力推进结构性改革，通过去产能、去库存、去杠杆、降成本、补短板，提高供给体系质量和效益，增强持续增长动力。二是更加注重改革创新，激发发展活力。围绕全面深化改革，始终坚持问题导向，进一步深化重点领域和关键环节改革，不断释放改革红利。围绕建设创新型社会，大力推进科技创新、制度创新、管理创新、业态创新等各方面创新，以创新推动经济转型升级。三是更加注重开放升级，拓展发展空间。整合资源优势、产业优势、区位优势，主动对接国家、省重大区域发展战略，进一步创新开放模式、完善开放机制、强化开放合作，积极参与区域经济分工协作，以区域联动助推发展升级。四是更加注重城乡统筹，促进协调发展。依托国家新型城镇化综合试点，突出城乡规划管理、基础设施、公共服务“三个一体化”，有序推进以人为核心的新型城镇化进程，走出具有鹰潭特色的新型城镇化道路，打造经济社会发展新引擎。五是更加注重改善民生，推进共建共享。坚持把保障改善民生放在更加重要位置，大力实施精准扶贫、精准脱贫，补齐民生短板保障公共服务，突出绿色发展优化生态环境，使人民群众有更多获得感。

具体来说，重点抓好以下七个方面的工作：

(一)坚持多点发力，推动经济稳定增长。

把稳增长放在首要位置，着力在挖潜能扩投入、兴实体强动能上下功夫，继续保持经济发展的良好态势。

1.强化投资的关键作用。加快项目建设，坚持以“8+1”重点工作为统领，全年安排大中型建设项目292个，总投资955亿元，年计划投资336亿元。其中，新开工建设皖赣线电气化改造等123个项目，建成投产新希望30万吨饲料加工等63个项目。创新投资方式，做大做强四大市级融资平台，推动有轨电车等总投资336亿元的47个PPP项目签约建设，加强政府购买服务项目支出管理，扩大政府购买服务范围。加大“三争”力度，紧密对接国家“七大工程包”、“六大消费工程”和四类新工程包等投资领域，积极争取国家专项建设基金，做好花桥水利枢纽等项目前期工作，加快实施一批重大项目，不断增强发展后劲。

2.强化消费的基础作用。促进信息消费，加快新一代信息基础设施建设，大力推广电子商务，推进鹰潭电商创新孵化基地、贵溪电商生态产业园、余江电商创业园等十大电商产业园建设，大力发展阿里巴巴余江农村淘宝，加快形成线上线下联通互促的新商业模式和消费方式，力争电子商务交易额突破400亿元。稳定住房消费，推进以满足新市民住房需求为出发点的住房制度改革，打通商品房与棚改安置房、公租房、租赁房三个渠道，发展住房租赁市场，实现公租房货币化，有效释放住房刚性需求和改善性需求，着力化解房地产库存。发展文化消费，扶持国际眼镜城等综合体健康发展，推进文化创意小镇建设，打造一批特色街区，举办眼镜、黄蜡石等区域性展销会，加强文化产业公共服务平台建设，着力打造文化创意产业集聚区。推动旅游消费，落实带薪休假制度，积极发展农家乐、乡村度假、户外运动等休闲旅游新业态。大力发展养老、家政、健康消费，满足多层次、多样化需求。

3.强化企业的主体作用。健全企业帮扶机制，加强政策引导服务，着力帮助企业解难题、促发展。深入开展降低实体经济企业成本行动，积极落实结构性减税、普遍性降费等政策，实施涉企收费目录清单管理，降低企业交易、财务、物流、人工、用电等成本，切实减轻企业负担。积极清理“僵尸企业”，优化、消化存量产能，力争淘汰落后铜产能20万吨。深化校企对接合作，加强技能培训，拓宽招工渠道，破解企业用工难题。

4.强化金融的支撑作用。整合财政性资金统筹设立市级产业发展基金，重点扶持产业发展升级。强化市工业控股公司和市中小企业信用担保有限公司的运作，推广“财园信贷通、财政惠农信贷通”等融资模式，整合组建市融资担保公司，引导更多资金投向实体经济。发展普惠金融，力争兴业银行等股份制银行在我市设立分支机构，推动民间融资登记服务机构稳健经营。加大企业直接融资力度，力争新增上市企业1家、上“新三板”企业3家。

(二)坚持三产融合，加快产业升级步伐。

以新型工业化为核心，协同推进现代服务业和现代农业加快发展，力促在做大总量、调优结构、彰显特色等方面取得突破，进一步提升产业发展的质量和效益。

1.深入实施工业强市战略。以沿320国道产业集聚升级带为载体，大力实施“工业强基、企业减负、产业升级”工程，力争规模以上工业主营业务收入突破 3450亿元，市属工业、铜精深加工、非铜产业比重分别提高2、7、2个百分点。以铜产业转型升级为主导，推进胜华22万吨铜杆等6个项目建设，新增铜精深加工产能32.5万吨。依托铜期货交割库和中江国际商品交易中心，大力开展铜贸易招商，积极争取设立国家物资储备库，力争全年铜贸易额突破500亿元。健全铜拆解加工区管理机制，创新海关、检验检疫服务方式，力争全年进口废料增长50%以上。以培育特色产业集群为方向，推进以绿色水工、机械装备为主导的智能制造业，加快一芯科技1.8亿张智能芯片等项目建设，支持三川集团发展“智慧水务+绿色水工+健康水业”，大力引进配套关联企业，建成全国重要的现代水工产业基地。推进以LED照明和新能源为主的光电新能源产业，确保阳光照明5000万套LED节能灯等项目尽快达产，加快中节能贵溪光伏发电站等新能源项目建设，争取装机容量达180兆瓦。推进以绿色食品和饮料为主的大健康产业，加快云露食品等项目建设，确保养元“六个核桃”等项目达产达标。推进贵溪发展精细化工产业、余江发展创意制造产业。以园区产城融合为支撑，落实好支持园区发展的各项政策，加快小微企业孵化基地二期、园区道路管网、保障性住房等基础设施建设，推进新市民保障工程，不断提升园区承载能力，力争龙岗工业园创建省级产业园，支持贵溪经开区创建国家级经济技术开发区，余江工业园扩区4平方公里。

2.加快发展现代服务业。着力加快旅游城市建设。按照“连点串线、扩面成片”的思路，确保圣井山综合开发、逍遥城综合体、智慧龙虎山体验中心等项目建成运营，推进龙虎山旅游产业园、天师林等项目建设，加快白鹤湖生态休闲观光项目开发，大力发展乡村旅游，努力形成多极支撑、南呼北应、城景一体的大旅游发展格局，确保圆满承办全省旅游发展大会。按照建设旅游城市的要求，规划建设休闲街区、城市绿道、骑行公园和慢行系统，加快推进城区主要道路和公共场所双语标识设置，确保龙虎山连接线改造和天洪公路竣工通车。力争全市接待国内游客增长28%、旅游总收入增长35%。着力打造区域物流中心。依托物流龙头企业，整合零散物流信息网点、零担快递网点，建成集信息、配货、仓储、中转服务功能为一体的第四方物流综合服务平台。加快现代物流园和国际商贸园建设，推动浙商联盟建材家居城等专业市场规范运营，力促大三江电商物流港等一批项目建成投运，形成物流集聚发展效应。

3.大力推进农业现代化。加强现代农业基础设施建设，整合涉农资金，加大农业投入，提高农业综合生产能力。大力实施藏粮于地、藏粮于技战略，新增高标准农田5万亩。加快推进农业产业化进程，重点发展绿色大米生产加工、优质特色果业等八大农业产业工程，加快发展苗木花卉、毛竹等六大林业特色产业。积极发展农业“接二连三”新业态，推广农业新技术、新品种，新增“三品一标”认证企业15个。积极培育新型农业经营主体，力争新增市级以上农业龙头企业20家以上，销售收入100亿元以上，新增农民专业合作社300家、家庭农场120家。加快白鹤湖现代农业示范园区建设，开工建设滨湖生态游憩区、农业科技园等项目，建成白鹤湖大道、苗木花卉景观带，引进落户一批优质现代农业项目，确保示范园区生态建设与环境保护协调发展。

(三)坚持城乡统筹，加速新型城镇化建设。

按照中央城市工作会议“五个三”的新要求，坚持以人为核心，以城乡一体发展为引领，扎实推进国家新型城镇化综合试点建设，使城镇功能不断完善，城市品质明显提升，城乡环境更加宜居。

1.以市域规划为龙头，优化城市空间布局。争取市城乡总体规划暨“多规合一”(2014—2030年)顺利获批，修编完善土地利用规划，加快编制生态环保规划，建成“多规合一”信息管理平台，实现“一张蓝图”管控城乡空间。按照“一核两带四区”空间布局，推进320国道拓宽改造、余信贵大桥、余信贵大道贵溪段和余江段建设，启动信江南路延伸段项目，推动贵溪主城区、余江城东新区、月湖新城开发建设，促进鹰贵、鹰余同城化相向发展。

2.以信江新区为核心，增强中心城区承载力。加快信江新区繁荣发展步伐。强化商业功能，加快推进天虹商场等商业综合体建设，繁荣发展信江大市场和步行街。强化公共服务功能，确保高教园区春季开学，恒大学校秋季开学，加快新二中、中小学生示范性综合实践基地建设，引进战略合作商托管运营人民医院信江新院，开工建设“四馆”工程。强化市政配套功能，开工建设信江新区北区总长11公里的7条道路，建成信江新区菜市场、垃圾转运站、污水处理厂。加强城市地上和地下基础设施建设。加大棚户区和城中村改造力度，推进老一中、二二三地质队等片区改造，启动防腐厂、东湖等片区改造，完成8.5万平方米改造任务。推进城市地下综合管廊和海绵城市建设，加快林荫西路改造、院里路贯通等项目建设，实施主城区75公里的雨污水管网、燃气管网改造，不断完善城市功能。

3.以精细管理为重点，不断提升城市品位。推进城市网络化、信息化、数字化管理，加快实施智慧城市和信息惠民国家双试点工程，完成城市公共信息服务平台、云计算中心等项目建设。加强城市公共管理，实施城区道路临时占道停车收费经营，加快城区停车场以及主城区公共自行车系统建设，优化公交运营线路。加大城市环境治理力度，开展城区建筑垃圾、扬尘污染专项整治，营造干净整洁有序的市容环境，积极争创省级文明城市。

4.以城乡一体为方向，促进城乡统筹发展。改革完善户籍制度，实施居住证制度，推动城镇常住人口基本公共服务均等化，有序推进进城农民转移落户。加快镇村联动建设步伐，着力提升中心镇的产业集聚和公共服务水平，十强中心镇污水处理厂全部开工，建成塘湾、锦江、上清3个污水处理厂，完成200个左右“秀美乡村”建设点任务。建成市生活垃圾焚烧发电厂，启用农村11座垃圾中转站，实施城乡生活垃圾委托第三方治理，逐步推行“户分类、村收集、镇转运、市处理”城乡环卫一体化模式。着力推进城乡交通客运一体化建设，加快鹰北公路客运枢纽站和公交枢纽站、鹰西短途客运站、鹰南公交枢纽站建设，升级改造省道 41.6公里，完成县乡道升级改造100公里、通自然村公路硬化240公里、公路危桥改造加固15座，推动城市公共服务向农村延伸。

(四)坚持改革创新，增强发展动力活力。

牢牢把握新常态下的新形势，着力强化改革、开放和创新三大动力支撑，努力开创体制机制更活、发展环境更优、发展动力更足的新局面。

1.加大改革攻坚力度。抓好改革举措落实。推进简政放权，加强行政审批目录管理，完善行政权责清单动态调整机制，深化政务服务网建设，规范运营“中介超市”。深化财税改革，扩大预算绩效管理范围，加大财政资金统筹使用力度，实行政府债务余额限额管理，加快综合治税平台和非税收入征缴系统建设。加大企业信用监管警示系统的应用力度，加强“信用鹰潭”体系建设。全面完成农村土地承包经营权确权登记颁证工作，加快农村综合产权交易市场建设，开展农村集体产权制度改革试点，推进供销合作社和农垦改革，扩大政策性涉农保险范围。稳步推进公务用车改革。深化不动产统一登记改革工作。推进事业单位分类改革。加快绿色殡葬改革。大力推进先行先试。积极推进余江国家农村宅基地制度改革试点，形成制度性成果和经验。推进全国行政综合执法改革试点，推进跨部门、跨行业综合执法。推进贵溪、余江全国深化县城基础设施投融资体制改革试点，设立新型城镇化产业发展基金。积极开展科技金融试点工作。积极探索特色改革。探索高新区市场化运作机制，加快产城融合发展。推进国家级旅游业改革创新先行区建设，推动龙虎山文化旅游发展集团引入战略合作伙伴，搭建盘活旅游资产、整合旅游要素、开发旅游产品新平台。完善国有资产管理体制，健全国有资本投资运营机制。

2.加大开放升级力度。融入“一体化”格局。高位对接长江经济带、沪昆高铁经济带等重大区域战略，积极推动鹰梅铁路、鹰潭机场、贵溪—资溪高速公路、信江河道整治等项目落地，在市场要素整合、产业分工协作、公共信息共享等领域找准共同对接点，着力分享重大区域战略带来的发展红利。提升“引进来”成效。策应全省招商引资重大项目攻坚行动，加强重点产业招商，更加重视引进技术、管理等高端生产要素，提高招商引资质量水平。拓展民企入赣成果，实施“鹰商回归”提升工程，力争全年引进省外5000万元以上项目资金311亿元，实际利用外资2.6亿美元。加快“走出去”步伐。抓住我市列入全省国际贸易“单一口岸”试点的有利时机，加快口岸公共信息平台建设，实现单一窗口和通关一体化。鼓励有条件的企业“走出去”，抢占更多国际市场份额，力争实现外贸出口 9.48亿美元。

3.加大创新创业力度。搭建好创新创业平台。加快发展众创空间、创新工场等新型创业孵化模式，带动一批创客团队、创客平台快速集聚发展。推动铜检中心和铜产业工程技术中心整合，力争铜检中心取得国家实验室资质。培育好创新创业主体。积极策应全省创新驱动“5511”工程，推进“两化”融合，深入实施 “互联网+”智能制造行动计划，推进国家知名品牌创建示范区建设，新认定高新技术企业10家以上，高新技术产业产值增长15%。营造好创新创业氛围。深化商事制度改革，进一步放宽民间投资准入领域，促进非公有制经济发展。制定更加开放、更加灵活的人才政策，大力培养和引进创新人才。落实创业各项奖励扶持政策，大力扶持创业活动和新业态培育，努力营造尊重、保护创新创业的良好环境。

(五)坚持绿色发展，推进生态文明建设。

以创建生态文明先行示范区为契机，坚持绿色崛起、绿色富民的价值取向，注重绿色生产和消费，打响生态品牌，走出一条绿色、低碳、循环发展道路。

1.加强环境保护治理。深入实施大气、水、土壤污染防治行动计划，完善大气环境质量监测预警体系，力争环境空气质量优良率达到80%以上。推进水生态文明建设，全面实施“河长制”，启动农村饮水提升增效和山塘整治工作，推进全国小农水重点县、信江河治理、童家联圩等项目，实施中小型水库水质治理、畜禽清洁生产和标准化建设。加强水源监测和日常监管，确保集中式饮用水水源地水质达标率100%。推动六国化工废水综合治理项目，推进海利化工、麒麟化工搬迁入园。继续实施贵冶周边土壤修复工程，强化企业固体废物分类处理，严格危险废物管理。

2.大力发展绿色经济。实施工业绿色发展行动计划，推进国家工业绿色转型发展试点城市、国家第二批资源综合利用“双百工程”示范基地和国家循环化改造试点园区建设，加大贵冶余热综合利用项目推进力度。大力发展生态农业，深入推进农业标准化、清洁化生产，打造一批以“道教龙虎山、绿色农产品”为主题的知名品牌。加速提升生产性服务业、旅游业和新兴服务业发展，着力构建集约高效的绿色服务业体系。积极推行绿色建筑，促进建筑产业优化升级。完善节约集约用地制度和评价体系，开展批而未供和闲置土地新一轮清理工作。

3.推进绿色生态建设。实施人工造林4.4万亩，加快低产低效林改造和森林抚育步伐，重点保护好“一区两园”，建成鹰西湿地公园、信江生态公园、信江植物园，加快白露河治理及景观提升改造，力争成功创建国家森林城市。强化生态文明制度建设，落实领导干部任期生态文明建设责任制、自然资源资产离任审计制度和生态环境损害责任终身追究制。加大环境执法力度，严厉惩处违法违规行为。

(六)坚持惠民共享，增强人民群众获得感。

按照“人人参与、人人尽力、人人享有”的要求，坚守底线、突出重点、完善制度、引导预期，切实增强人民群众的获得感。

1.倾力办好民生实事。办好十大惠民共享工程70件实事，确保全年一般公共预算支出中民生支出比重达70%。深入实施大学生就业促进计划和创业引领计划，进一步加强对贫困家庭劳动力、农业富余劳动力等就业困难群体帮扶，努力提升公共就业服务能力。完善“多险合一”信息系统功能，健全基本养老、基本医疗、城乡低保等制度，建立机关事业单位职业年金制度。

2.大力推进脱贫攻坚。实施精准扶贫、精准脱贫，建立更加精准系统的扶贫台账。抓好产业扶贫，采取“合作社+基地+农户”等多种形式，建立完善产业发展与贫困群众之间的利益联结机制，形成贫困村“一村一品”特色产业。抓好保障扶贫，拓宽扶贫资金来源渠道，健全农村低保制度，加强医疗保险和医疗救助，加快实施教育扶贫工程，加大贫困地区基础设施投入力度，力争退出贫困人口8628人、退出贫困村7个。

3.协调发展社会事业。统筹推进学前教育、义务教育均衡发展，加快全省教育信息化试点建设，提高优质教育资源覆盖面。完成“三校合一”整合。推进健康鹰潭建设，全面实施国家城市公立医院综合改革，推进分级诊疗制度建设，着力解决“看病难、看病贵”问题。实施全面两孩政策，合理配置妇幼保健、学前和中小学教育、社会保障等资源，满足新增公共服务需求。大力发展群众体育和竞技体育。做好大上清宫遗址现场考古发掘工作。抓好豆制品和米粉集中加工区建设。提升市综合检验检测中心能力，建立全程监管体系，确保食品药品安全。深入推进军民融合发展，加强国防教育、人防民防、民兵预备役建设。进一步做好统计、档案方志、民族宗教、外事侨务、涉台事务、气象、防灾减灾、老龄和残疾人等工作，支持各民主党派、工商联、知识分子联谊会和工会、共青团、妇联等人民团体的工作。

4.提高社会治理水平。深入推进法治鹰潭建设，启动实施“七五”普法规划，健全人民调解、行政调解、司法调解联动的衔接配合机制，推动城乡律师法律服务均等化。

完善“12345”政务服务热线运行机制，打造阳光信访，依法化解信访突出问题。深入推进平安鹰潭建设，完善打防控一体的社会治安防控体系，建成并运行“天网”工程，依法打击各类违法犯罪行为，增强人民群众安全感。落实“党政同责、一岗双责、失职追责”制度，狠抓安全生产工作，提升应急处置水平，防范和遏制重特大安全事故。

(七)坚持务实进取，切实加强政府自身建设。

“四个全面”战略布局，对新常态下做好政府工作提出了新的更高要求。我们必须解放思想、敢于创新、勇于担当，做到换届之年思想不懈怠、工作不松劲，确保“十三五”开好局、起好步。

1.围绕全面建成小康社会，进一步强化发展意识。面对新形势，我们将始终牢记发展是第一要务，增强对“五个发展”的统筹能力，不折不扣落实中央、省委省政府和市委的重大决策部署，以创造性工作把决策部署转化为实实在在的发展成效。面对新常态，我们将始终秉承“环境是竞争力，也是生产力”的理念，进一步提升服务能力和水平，加快服务型政府建设，着力营造更加开放包容的发展环境。面对新任务，我们将始终保持“一着不让抓落实”的紧迫感和责任感，加强对重大决策部署落实情况的督促检查，加强政府部门目标责任制考核，确保完成全年目标任务。

2.围绕全面深化改革，进一步强化担当意识。改革进入攻坚克难深水区，决心比认识更重要，担当比方法更重要。做好今年工作，必须大胆改革、锐意创新。我们将坚持把改革创新精神融入到政府工作各个方面，贯穿于全年工作始终，勇于面对矛盾、直视问题，敢于攻坚闯关、破解难题，善于创新举措、探索新路，以关键领域、重点环节的突破，进一步累积叠加鹰潭发展的后发优势。

3.围绕全面依法治国，进一步强化法治意识。加快法治政府建设，强化法治思维，健全法治政府考核评价指标体系，提高依法行政、依法办事的能力和水平。完善依法决策机制，普遍建立政府法律顾问制度，推进决策的科学化、民主化、法制化。加强行政执法队伍建设，严格规范公正文明执法，提高行政执法水平。自觉接受市人大法律监督、市政协民主监督和社会公众舆论监督，高效办理人大代表建议和政协提案。

4.围绕全面从严治党，进一步强化廉洁意识。巩固和拓展党的群众路线教育实践活动和“三严三实”专题教育成果，坚持把纪律和规矩挺在前面，严格落实 “一准则两条例”，严格执行中央八项规定，坚决反对“四风”，遏制不作为、乱作为的懒政怠政行为，推动作风建设常态化。坚持用制度管权管事管人，严格落实党风廉政建设“两个责任”，深入推进惩治和预防腐败体系建设，切实加强重点领域和关键环节监管，营造风清气正的政治生态。

各位代表!“十三五”的蓝图已经绘就，新一轮发展的号角已经吹响。让我们紧密团结在以习近平同志为总书记的党中央周围，在市委的坚强领导下，勇于担当、开拓奋进、不负重托，确保实现“十三五”良好开局，奋力谱写建设富裕秀美宜居和谐鹰潭、全面建成小康社会的新篇章!
